# A framework for accelerated phototrophic bioprocess development: integration of parallelized microscale cultivation, laboratory automation and Kriging-assisted experimental design

**DOI:** 10.1186/s13068-017-0711-6

**Published:** 2017-01-31

**Authors:** Holger Morschett, Lars Freier, Jannis Rohde, Wolfgang Wiechert, Eric von Lieres, Marco Oldiges

**Affiliations:** 1 Forschungszentrum Jülich GmbH, Institute of Bio- and Geosciences, IBG-1: Biotechnology, Wilhelm-Johnen-Straße, 52428 Jülich, Germany; 2 Institute of Biotechnology, RWTH Aachen University, Aachen, Germany

**Keywords:** Biodiesel, *Chlorella vulgaris*, Design of experiments, Kriging, Lipid production

## Abstract

**Background:**

Even though microalgae-derived biodiesel has regained interest within the last decade, industrial production is still challenging for economic reasons. Besides reactor design, as well as value chain and strain engineering, laborious and slow early-stage parameter optimization represents a major drawback.

**Results:**

The present study introduces a framework for the accelerated development of phototrophic bioprocesses. A state-of-the-art micro-photobioreactor supported by a liquid-handling robot for automated medium preparation and product quantification was used. To take full advantage of the technology’s experimental capacity, Kriging-assisted experimental design was integrated to enable highly efficient execution of screening applications. The resulting platform was used for medium optimization of a lipid production process using *Chlorella vulgaris* toward maximum volumetric productivity. Within only four experimental rounds, lipid production was increased approximately threefold to 212 ± 11 mg L^−1^ d^−1^. Besides nitrogen availability as a key parameter, magnesium, calcium and various trace elements were shown to be of crucial importance. Here, synergistic multi-parameter interactions as revealed by the experimental design introduced significant further optimization potential.

**Conclusions:**

The integration of parallelized microscale cultivation, laboratory automation and Kriging-assisted experimental design proved to be a fruitful tool for the accelerated development of phototrophic bioprocesses. By means of the proposed technology, the targeted optimization task was conducted in a very timely and material-efficient manner.

**Electronic supplementary material:**

The online version of this article (doi:10.1186/s13068-017-0711-6) contains supplementary material, which is available to authorized users.

## Background

By virtue of significant advantages offered over agricultural crops [1–6], microalgae are generally accepted as promising feedstock for bio-economy applications [7–9]. However, until now, their industrial exploitation remains mostly uneconomic, especially when lower-priced products like biofuels are targeted [10]. Currently, the integrated utilization of biomass is intensively investigated as a promising concept to improve the overall efficiency in terms of cost and energy [9, 11, 12]. In this context, intracellular lipids represent a compound class of special interest as they can be either transesterificated to biodiesel [7] or boost the nutritional quality of algae for functional food applications [13].

Regarding phototrophic bioprocess development, early-stage strain and parameter screening are of crucial importance to the successful set-up of economic processes [14]. Today, these aspects are typically studied by means of only marginally parallelized reactor systems like shake flasks, test tubes or even single-vessel reactors [15–18]. Consequently, experimental throughput is fairly limited rendering screening tasks rather laborious and highly time consuming. Only recently a strong demand for high throughput micro-photobioreactors has been identified, based on which some prototype systems have been developed [19–27]. To take full advantage of phototrophic microscale cultivation, supporting methodologies and technologies, such as simplified strain maintenance [28], high throughput analytics [29, 30], and automated processing [20, 31], are needed.

In the medium term, these initial developments and especially further progress in high throughput technology and laboratory automation will clearly boost the efficiency of phototrophic process development. Phototrophic processes are characterized by their intrinsic complexity induced by a high number of potentially interacting input variables. Hence, experimental capacities, i.e., mainly cultivation, will be always a crucial factor due to the trade-off between throughput and the necessary laboratory resources. Current micro-photobioreactors mainly rely on standardized microtiter plates. Thus, a further rise of cultivation capacity by intensifying parallelization would need to be based on scale out and inevitably be accompanied by increasing cost. Hence, an alternative strategy to focus cultivation activities on only the most informative experiments is the ultimate solution to tackle the omnipresent challenge of restricted experimental throughput.

One approach to achieve this efficiently, which is already well-established for microbial bioprocess development [32], is the use of Design of Experiments (DoE) to focus on experiments providing the highest information content in a targeted parameter space. Despite having been established during the early twentieth century [33], there is still ongoing research into this methodology [34]. This approach is regarded to be particularly suitable to deal with the combinatorial explosion typically occurring when investigating multi-parameter relations [35]. Moreover, DoE overcomes a critical limitation of “conventional” one-factor-at-a-time experiments, as such approaches often fail in locating global optima by not taking potentially synergistic or antagonistic interactions of input variables into account [36]. Regarding bioprocess development, the most prominent application of DoE is the culture media optimization [32]. During such tasks, the omnipresent interactions between single compounds render locating a global optimum by “conventional” experimental planning to chance.

In the above context, the current study aims at the combination of emerging technologies for parallelized microscale cultivation and analytics to phototrophic microorganisms with elaborate experimental design as has previously been fruitfully applied for heterotrophic systems by [37]. Thereby, an integrated framework for the accelerated development of phototrophic bioprocesses is to be set up. Optimizing medium composition toward maximized lipid productivity of the unicellular microalga *Chlorella vulgaris* was chosen as a model process for the above purpose.

## Methods

### Chemicals, strain

All chemicals were purchased either from Sigma-Aldrich (Steinheim/Germany) or Roth (Karlsruhe/Germany) and were of analytical grade. The unicellular microalga *C. vulgaris* 211-11b [38], purchased from the Culture Collection of Algae at the University of Göttingen (Germany), was used throughout all cultivation experiments.

### Medium

Cultivations were carried out in variations of an enriched Bold’s Basal Medium [39] prepared from stock solutions. The previously established reference medium [27, 28] was composed of chemicals as follows: 9.76 g L^−1^ 2-(*N*-morpholino)ethanesulfonic acid (MES), 0.6 g L^−1^ K_2_HPO_4_, 1.4 g L^−1^ KH_2_PO_4_, 1.5 g L^−1^ NaNO_3_, 187.5 mg L^−1^ MgSO_4_·7 H_2_O, 6.25 mg L^−1^ NaCl, 125 mg L^−1^ CaCl_2_·2 H_2_O, 17.64 mg L^−1^ ZnSO_4_·7 H_2_O, 2.88 mg L^−1^ MnCl_2_·4 H_2_O, 2.4 mg L^−1^ Na_2_MoO_4_·2 H_2_O, 3.14 mg L^−1^ CuSO_4_·5 H_2_O, 0.94 mg L^−1^ CoSO_4_·7 H_2_O, 22.8 mg L^−1^ H_3_BO_3_, 9.96 mg L^−1^ FeSO_4_·7 H_2_O, 3.68 mg L^−1^ H_2_SO_4_, 100 mg L^−1^ Na_2_EDTA·2 H_2_O, 62 mg L^−1^ KOH and 100 mg L^−1^ penicillin-G sodium salt. The pH value was set to 6.5 with 5 M NaOH. Ultrapure water (type 1) was used for the preparation of all cultivation media.

During optimization experiments, the medium composition was varied according to the respective experimental plan by adjusting the applied volumes of the individual stock solutions. These media variants were prepared by a liquid-handling platform as previously described in literature. Medium preparation was carried out in a fully automated manner, while a surrounding laminar flow hood ensured sterile conditions [34, 40, 41]. Media were prepared at 2.5 mL scale in an MTP-R-48-B “Round Well Plate” (m2p-labs, Baesweiler/Germany) under continuous shaking at 500 rpm on an integrated Teleshake 95 (Inheco, Martinsried/Germany), while the minimum volume to be pipetted was set to 10 µL. Thereby, achieving sufficiently high accuracy (±0.3%) and precision (±0.3%) could be ensured [34]. Subsequently, 950 µL of each medium was transferred to a well of an MTP-48-B “FlowerPlate^®^” (m2p-labs, Baesweiler/Germany) in which the cultivation took place (see “Main cultivation” section).

### Strain maintenance and pre-cultivation

*Chlorella vulgaris* was maintained as glucose-adapted cryocultures. Preserved cells were re-adapted to light during phototrophic pre-cultivation in illuminated shake flasks. A detailed description of strain maintenance and pre-cultivation strategy can be obtained from [28]. After 60 h of incubation, the cells were harvested by 5 min centrifugation at 3939×*g* and 4 °C in a Labofuge 400R (Heraeus Instruments, Hanau/Germany). The supernatant was discarded, and the pellet re-suspended in 0.9% (w v^−1^) NaCl to a biovolume of 2 µL mL^−1^ to generate the stock solution required for the inoculation of subsequent main cultivations.

### Main cultivation

Main cultivations were conducted in pre-sterilized, disposable 48-well MTP-48-B “FlowerPlates^®^” (m2p-labs, Baesweiler/Germany). Each well was filled with 950 µL of medium and inoculated to a biovolume of 0.1 µL mL^−1^ with 50 µL of the inoculation stock solution generated as described in “Strain maintenance and pre-cultivation” section. The plates were sealed using an F-R48-10 “perforated sealing foil for evaporation reduction” (m2p-labs, Baesweiler/Germany), pasted over with an F-GP-AB10 “gas-permeable seal” (m2p-labs, Baesweiler/Germany).

The microtiter plates were incubated using a micro-photobioreactor prototype. The system relies on bottom-side illumination with a set of blue and white LEDs and indirect temperature control via placement of the plates in a tempered incubation chamber. A detailed description and the schematic representation of the system are given in [27]. The following cultivation conditions were applied: 25 °C, continuous shaking at 1200 rpm, 3 mm shaking diameter, 2.5% (v v^−1^) CO_2_, 200 µmol m^−2^ s^−1^ photon flux density (constant), and ≥85% relative humidity.

### Biomass detection

Optical density (OD) was acquired using 10-mm polystyrene semi-micro cuvettes (ratiolab, Dreieich/Germany) and an UV-1800 photometer (Shimadzu, Duisburg/Germany) at 750 nm, while desalted water served as a blank. If needed, samples were diluted to OD_750_ ≤ 0.3 using 0.9% (w v^−1^) NaCl solution to fit the linear range of the photometer.

The biovolume was measured taking advantage of a particle counter (MultiSizer 3, Beckman Coulter, Krefeld/Germany) using the “Coulter principle” [42]. The device was equipped with a 30 µm capillary which had been calibrated using a suspension of 3 µm latex beads (Beckman Coulter, Krefeld/Germany) according to the manufacturer’s specification and was operated in volumetric control mode. Prior to measurement, cell suspensions were diluted to OD_750_ ≤ 0.025 in CASYton buffer (Omni Life Science, Bremen/Germany), and only particles in the range of 1.8–14 µm were analyzed.

The cell dry weight was determined by means of gravimetry. Culture liquid from two replicate wells of a microtiter plate was pooled to obtain sufficient sample amounts for the analysis. Cells were spun down in pre-dried and weighed 2-mL reaction tubes for 5 min at 16,060×*g* (Biofuge Pico, Heraeus Instruments, Hanau/Germany). The supernatants were discarded and the pellets freeze-dried in an LT-105 freeze dryer (Christ Gefriertrocknungsanlagen, Osterode am Harz/Germany) until attaining a constant weight. After acclimatization to room temperature in a desiccator, weighing was repeated, and the cell dry weight was derived from the resulting mass difference.

### Lipid quantificati**o**n

The intracellular accumulation of neutral lipids was quantitatively monitored by means of an automated high throughput Nile red staining assay as previously described in [29].

### Nitrate quantification

Cells were removed by filtration using 0.2 µm cellulose acetate syringe filters (DIA-Nielsen, Düren/Germany), and the cell-free supernatant was stored at −20 °C prior to analysis, if needed. Nitrate was quantified using the Spectroquant 1.09713.0002 nitrate test (Merck, Darmstadt/Germany) according to the manufacturer’s specifications, scaled down to one quarter of the recommended volume. Supernatants were pre-diluted with desalted water to fit the linear range of the assay, if needed. The measurements were conducted in UV semi-micro cuvettes (Brand, Wertheim/Germany) using an UV-1800 photometer (Shimadzu, Duisburg/Germany).

### Acquisition of fatty acid fingerprints

Lyophilized biomass from cell dry weight determination (see Sect. 2.5) was in-situ transesterificated using acidic methanol [10% (w w^−1^) H_2_SO_4_], and the resulting fatty acid methyl esters were subsequently extracted with heptane. Semi-quantitative fingerprints were accessed by gas chromatography time-of-flight mass spectrometry of the extracts. A detailed description of the methodology can be obtained from [43].

### Experimental design

Media composition was optimized with respect to lipid productivity using a Design of Experiments methodology. The applied optimization strategy was adopted from [34]. Initially, fractional and full factorial experimental designs were applied for estimating single component effects and combinatorial interactions. Myers et al. [44] provide a good overview of these classical DoE methods.

Based on the initially collected data, the statistically more advanced concept of Kriging was applied for data analysis, visualization, and for designing further experiments with potentially improved lipid productivity. Kriging is an interpolation method that provides unbiased approximations of the underlying nonlinear functional relationships between media composition and lipid productivity with minimal prediction error. This method originates in geostatistics and has recently been adapted for optimizing biotechnology processes [34]. Further mathematical details of the Kriging method can be found in the monograph of Cressie [45]. The statistical analysis tools applied in this study are part of the open source Kriging toolkit “KriKit”, which can be freely downloaded at https://github.com/modsim/KriKit.

### Expected Improvement

Given a Kriging model of the current dataset, further experiments were designed to maximize the Expected Improvement (EI). This experimental design strategy seeks a compromise between maximizing lipid productivity and reducing prediction uncertainty of the Kriging approximation in relevant regions of the parameter space [46]. In a comparative study, EI has been found to outperform other sampling strategies in Kriging-based optimization [47].

In sequential optimization, new experiments are typically planned at maximal EI. Parallel experiments, as in the present study, are most efficiently planned by sampling from the EI distribution. In a non-deterministic sampling process, using the Markov Chain Monte Carlo (MCMC) method, new experiments are selected with probability proportional to their EI. Naturally, experiments with high EI are preferred over experiments with lower EI, which nonetheless have a reduced chance of being selected, while experiments with zero EI are strictly excluded. Freier et al. have demonstrated that MCMC sampling can significantly reduce the number of required experiments in process optimization [48]. In the present study, the Delay Rejection Adaptive Metropolis algorithm [49] was applied with a chain length of 10,000 elements, of which the first 1000 are discarded (burn in phase of the MCMC method).

## Results and discussion

### Choice of relevant media components

The medium targeted for optimization incorporates 17 different components (see “Medium”) with phosphate salts counted as one compound due to their pH-dependent equilibrium. This number is too high to efficiently perform the experimental study with a manageable number of experiments, since a full factorial design with two concentration levels would result in 2^17^ ≈ 130,000 experiments. In order to keep the number of components of interest, preselection was completed based on the literature information. Table 1 summarizes the known biological effects of the individual components. Penicillin-G concentration was kept constant under all conditions, and all trace elements were clustered to one single input variable as a similar effect on cultivation was expected. Sulfuric acid and potassium hydroxide had to be varied together with FeSO_4_ and Na_2_EDTA, respectively, as they were needed to keep the latter two components dissolved in their stock solutions. Thereby, the number of input variables was reduced by almost 50% from 17 to 9.

**Table 1:** Initial evaluation of the medium components’ potentials for the optimization of lipid productivity

Component	Evaluation	Reference	Variation
CaCl_2_	Versatile effector in plant cells; reported to be essential for induction of lipid synthesis	[50–52]	Yes
FeSO_4_	Influence on growth and lipid metabolism reported	[51, 53–55]	Yes
H_2_SO_4_	Sulfur supply ensured by sulfate anions from diverse other medium components; nevertheless varied as provided together with FeSO_4_ in one stock solution		Yes^b^
K_2_HPO_4_/KH_2_PO_4_	Essential phosphorus source (nucleic acid synthesis)	[54]	Yes
KOH	Potassium excess by phosphate salts; nevertheless varied as provided together with Na_2_EDTA in one stock solution		Yes^c^
MES	Trade-off between osmotic inhibition and buffer capacity; alkaline pH may inhibit cell cycle	[56]	Yes
MgSO_4_	Influence on growth and lipid production reported; effector of acetyl-CoA carboxylase, an essential enzyme during lipid biosynthesis; central atom of chlorophyll	[51, 57, 58]	Yes
NaCl	Reported to increase lipid production; excess may cause metabolic burden (ATP dependent sodium exporters) and thus inhibit growth	[59, 60]	Yes
Na_2_EDTA	Commonly used metal chelator; excess may cause growth repression due to ion depletion	[51, 55]	Yes
NaNO_3_	Essential nitrogen source (protein synthesis)	[54]	Yes
Penicillin-G	Support of long-time sterile conditions; not metabolized (data not shown)		No
Trace elements (CoSO_4_, CuSO_4_, H_3_BO_3_, MnCl_2_, Na_2_MoO_4_, ZnSO_4_)	Numerous studies about wastewater detoxification available, but only limited information concerning metabolism and lipid production; general pattern: little amounts essential, but high level cytotoxic (e.g., inhibition of photosynthesis); thus clustered to one input variable	[61–66]	Yes^a^

^a^All trace elements were clustered to one single input variable

^b^Varied together with FeSO_4_ as provided in one single stock solution

^c^Varied together with Na_2_EDTA as provided in one single stock solution

### Kriging-assisted optimization

#### Fractional factorial

Starting with the nine remaining media components of interest, a full factorial design would require 2^9^ ≈ 500 experiments. Making full use of 48-fold parallelized microtiter plate cultivation (see “Main cultivation” section), this leads to a total of 11 experimental runs, equivalent to 4 months of cultivation time. In 12-fold parallelized shake flasks, the experiments would even take 14 months. Yet, such time scales are clearly far from feasible, underlining the necessity to effectively reduce the experimental effort.

Fractional factorial designs allow the reduction of the number of experiments by estimating only single component effects and a subset of combinatorial effects [44]. The chosen design (see Additional file 1 for both, design and corresponding measurement data) comprises 37 experiments, five of which represent the reference point using the enBBM_ref_ medium (see Additional file 2 for medium composition). Taking reference points into account allows for the investigation of measurement noise and normalization. The other experiments allowed for a statistical analysis of the effect of single components, as well as the interaction with magnesium ions. The interaction with this divalent metal ion was analyzed, as it is reported to be an effector of the acetyl-CoA carboxylase, an enzyme essential for lipid biosynthesis responsible for the initial step of carbon dioxide fixation to malonyl-CoA (see Table 1). An overview of the functionality of this enzyme complex and its regulation is given by Ohlrogge and Browse [67]. Thus, any interactions with this input variable are of special interest with respect to product accumulation in the cells.

**Fig. 1:**
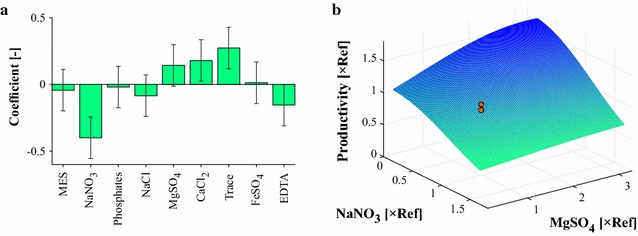
**a** Estimated relative effects of single components on lipid productivity. **b** Kriging visualization of combinatorial effect of MgSO_4_ and NaNO_3_ on lipid productivity. Remaining medium components are set to their reference values. *Red dots* indicate measurements at the reference point

Figure 1a shows the resulting statistical analysis of the fractional factorial experiments. The green bars indicate the expected effect of varying the medium concentrations between their minimal and maximal values (see Additional file 1). The error bars indicate the uncertainty of the estimations. In the following, the main and combinatorial effects of the components are checked for significance using a *t* test with a significance level of *p* = 0.1. Using a lower significance level would increase the risk of false negatives, i.e., excluding relevant components from the remaining study. The diagram shows that an increase in the concentration of NaNO_3_ has a significant negative effect on lipid productivity. On the other hand, an increase in the trace element’s concentration results in a significant (*p* < 0.1, *t* test) productivity improvement. Furthermore, the analysis indicates a positive tendency with the increasing CaCl_2_ concentration and the lowering EDTA concentration. However, because of measurement noise and the comparably low number of experiments, the uncertainty of the estimation is relatively high and leads to no reliable statement about the effects of CaCl_2_ and EDTA, respectively. Similarly, this holds true for MgSO_4_, but here, the pairwise interaction with another component was additionally investigated. As shown in Additional file 3, a significant negative combinatorial effect was identified with the sodium salts, NaNO_3_ and NaCl.

For visual inspection of the negative combinatorial effect, a Kriging model was constructed based on the given data. The predicted functional relationship between MgSO_4_, NaNO_3,_ and the lipid productivity is displayed in Fig. 1b. In case of low NaNO_3_ concentration, the interpolation reveals a positive correlation between an increase in MgSO_4_ and that of the performance indicator. With the increasing NaNO_3_ concentration, this positive effect is weakened.

In conclusion, significant effects of NaNO_3_ and the trace elements were identified, as well as positive tendencies of MgSO_4_ and CaCl_2_. Furthermore, the effect of MgSO_4_ appears to depend on the sodium salts, NaNO_3_ and NaCl. The remaining components have only low potential to affect the lipid productivity and were thus excluded from further analysis.

#### Full factorial

In order to verify the observed tendencies and to investigate potential pairwise or higher combinatorial effects, a full factorial design was constructed for the remaining five input variables: NaNO_3_, MgSO_4_, CaCl_2_, NaCl, and the clustered trace elements. This design again comprises five reference points and 32 experiments with minimal/maximal concentration (see Additional file 4 for the individual designs and the corresponding measurement data).

**Fig. 2:**
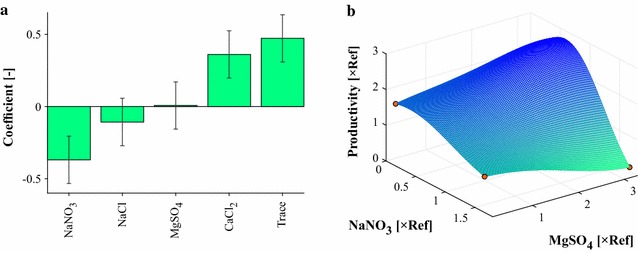
**a** Estimated relative effects of single components on lipid productivity. **b** Kriging visualization of the combinatorial effect of MgSO_4_ and NaNO_3_ on lipid productivity. NaCl was set to lower value, and CaCl_2_ and trace elements are set to upper values in the factorial design. *Red dots* indicate measurement data points

Figure 2a shows the updated statistical results after performing the full factorial design. The previously observed effects of NaNO_3_ and the trace elements were confirmed. The positive tendency of CaCl_2_ turned out to be significant, while the effect of NaCl remained insignificant. However, the interaction of MgSO_4_ with the sodium salts could be investigated in more detail. Figure 2b shows the opposing effect of MgSO_4_ dependent on NaNO_3_. This interaction leads to a non-distinguishable single component effect of MgSO_4_, as indicated in Fig. 2a. The analysis also revealed a negative interaction between CaCl_2_ and the trace elements, as indicated in the screening plot shown in Additional file 5.

#### Locating optimal medium composition

In “Full factorial” section, single and combinatorial effects of the media components were investigated on the basis of a full factorial design providing a rough estimate about optimal medium. The goal of the next step was to examine limitations of the particular effects and to identify potential optimal media compositions. To achieve this, the minimum and maximum concentrations were adjusted, and a more complex experimental design scheme was applied, comprising several nested factorial designs (see Additional file 6 for the full experimental design including the corresponding measurement data).

The maximum concentration of NaNO_3_ was lowered from 1.7 × Ref to 1 × Ref. The upper bound of the concentration of the clustered trace elements was increased by 50% to 3.75 × Ref. The concentration of CaCl_2_ could not be increased, as various types of precipitation effects were observed that distorted lipid analysis (see Additional file 7).

**Fig. 3:**
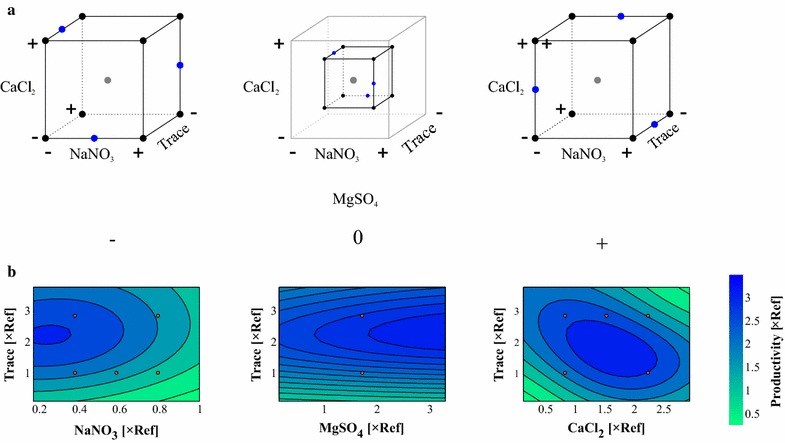
**a** Illustration of experimental design in the third round of experiments. *Black dots* represent full factorial designs with respect to NaNO_3_, CaCl_2_, and trace elements. *Blue dots* indicate additionally added points. *The gray dots* represent the respective center points. *Minus* and *plus signs* symbolize minimum and maximum concentrations of the respective components. **b** Contour plots of the Kriging interpolation. The non-varied components were fixed at MgSO_4_ = 1.7 × Ref, CaCl_2_ = 0.82 × Ref, Trace = 1 × Ref, or NaNO_3_ = 0.37 × Ref. *Red dots* indicate measurement data

However, MgSO_4_ was varied over three levels, as illustrated in Fig. 3a. For each level, the concentrations of NaNO_3_, CaCl_2_, and trace elements were distributed using a full factorial design. For the intermediate concentration of MgSO_4_, the remaining components were varied only over half of their total ranges. A center point was located in each of these full factorial cubes. An additional nine points were space filling distributed over the edges of the cubes. In total, 39 experiments were performed and analyzed, including four reference replicates.

Figure 3b shows the Kriging interpolation based on all the data available after the third round of experiments. The figure shows three contour plots where the third component was fixed to the front, bottom–left corner of the inner cube as shown in Fig. 3a. The contour plots clearly show an interaction of the trace elements with NaNO_3_ and CaCl_2_, whereas MgSO_4_ influences the lipid productivity only slightly positively. Moreover, an optimal region for the medium composition can be identified around MgSO_4_ = 3.25 × Ref, CaCl_2_ = 1.5 × Ref, Trace = 2 × Ref, and NaNO_3_ = 0.3 × Ref (A three-dimensional plot of the Kriging model together with the measured data can be obtained from Additional file 8).

#### Refining the optimum

In the fourth and last round of the experiments, twelve experiments were placed around the optimum predicted by the Kriging interpolation. These experiments were planned by sampling the EI distribution, as described in “Expected Improvement” section, for maximizing the lipid productivity and minimizing the prediction uncertainty of the Kriging model. In addition, 23 experiments were uniformly distributed over the parameter space in a random manner, in order to improve prediction accuracy also in non-optimal regions. In total, 39 experiments were performed, including the four reference experiments (The full experimental design including the respective measurement data can be obtained from Additional file 9).

**Fig. 4:**
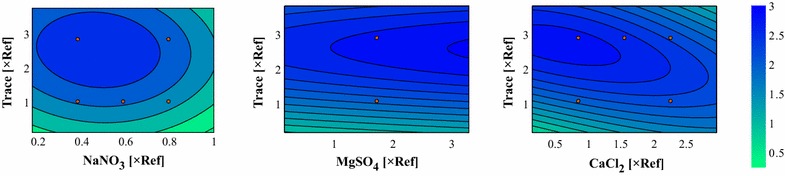
Contour plots of the updated Kriging interpolation after the fourth round of experiments. The non-varied components were fixed at MgSO_4_ = 1.7 × Ref, CaCl_2_ = 0.82 × Ref, Trace = 1 × Ref, or NaNO_3_ = 0.37 × Ref. *Red dots* indicate measurement data

Figure 4 shows predictions of the updated Kriging model in the same fashion as described in “Locating optimal medium composition” section. The location of the optimum shifted toward MgSO_4_ = 3.25 × Ref, CaCl_2_ = 1.25 × Ref, trace elements = 2.5 × Ref, and NaNO_3_ = 0.45 × Ref (A three-dimensional plot of the Kriging model together with the measured data can be obtained from Additional file 10). For the optimal medium composition, the Kriging model predicts an increase by a factor of 3.03 ± 0.81 in lipid productivity compared with the reference medium.

### Validating the optimal medium composition

**Fig. 5:**
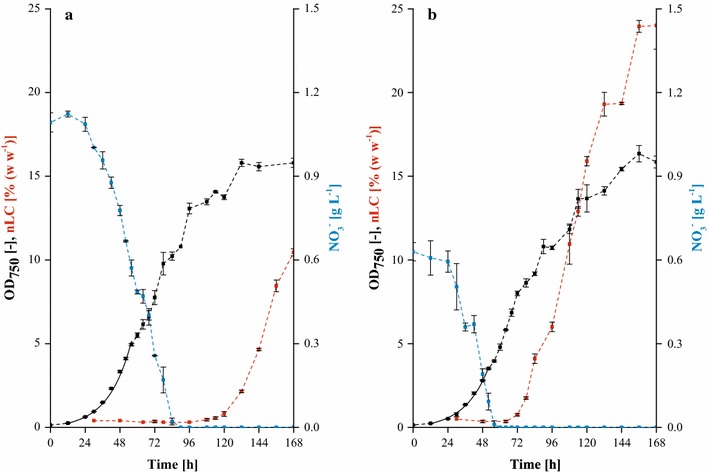
Comparison of the processes using reference and optimized medium. **a** enBBM_ref_, **b** enBBM_opt_; 25 °C, 2.5% (v v^−1^) CO_2_, 200 µmol m^−2^ s^−1^ PAR, ≥85% relative humidity. *Error bars* represent min/max from biological replicates (*n* = 2)

In order to validate the determined optimal medium composition (see “Kriging-assisted optimization” section) and to highlight potential changes of process kinetics, cultivations using enBBM_ref_ and enBBM_opt_ were carried out (see Additional file 2 for medium composition). Both processes were monitored in-depth by sequential harvest of replicate wells from microtiter plate cultivations (see Fig. 5). To maximize comparability with the literature reports, biomass concentration at harvest was acquired as cell dry weight rather than biovolume in this context.

Medium optimization resulted in a series of significant changes in process performance as summarized in Table 2. While the exponential growth rates in both media did not differ significantly (*p* < 0.05, *t* test), times to nitrogen depletion were 84 h and 52 h for enBBM_ref_ and enBBM_opt_, respectively. This was due to the reduction of nitrate concentration during medium optimization down to 0.45 × Ref. In the reference process, exponential growth shifted to linear kinetics reaching an optical density of 4.94 ± 0.06 typically indicating the onset of light limitation and in clear accordance with prior experiments [27]. This effect was not observed for the optimized medium before nitrogen depletion. Neutral lipid accumulations started within 36 h (enBBM_ref_) and 20 h (enBBM_opt_) after nitrogen limitation which corresponds to a reduction of approx. 45%. Moreover, the biomass-specific lipid accumulation rate (estimated by linear fit) increased by approx. 32% from 4.87 ± 0.53% (w w^−1^) d^−1^ to 6.43 ± 0.17% (w w^−1^) d^−1^ due to medium optimization. Most probably, both effects are attributable to the increased availability of magnesium and calcium ions, as well as trace elements in the medium. This might result in a boost of the enzymatic turnover of lipid synthesis, especially regarding acetyl-CoA carboxylase (see Table 1).

**Table 2:** Comparison of process performance indicators using reference and optimized media. Error bars represent min/max from biological replicates (*n* = 2)

Parameter	enBBM_ref_	enBBM_opt_
Exponential growth rate (d^−1^)	1.49 ± 0.06	1.45 ± 0.1
Time to nitrate depletion (h)	84	52
Delay from nitrate depletion to onset of lipid synthesis [h]	36	20
Biomass-specific lipid accumulation rate [% (w w^−1^) d^−1^]	4.87 ± 0.53	6.43 ± 0.17
Cell dry weight at harvest (g L^−1^)	4.95 ± 0.06	4.93 ± 0.01
Neutral lipid content at harvest [% (w w^−1^)]	10.55 ± 0.35	23.9 ± 1.2
Volumetric productivity (mg L^−1^ d^−1^)	74 ± 1	169 ± 7

Alternatively, a kinetic limitation of ion import into the cells at the low concentrations in the reference medium could be an explanation. Regarding downstream processing, the increased magnesium concentration offers another positive aspect, as it was previously reported to assist flocculation of the cells at high pH [68]. This mechanism is currently being investigated as an alternative to the comparably costlier biomass separation by centrifugation.

Most strikingly, cell dry weight at harvest did not differ significantly (*p* < 0.05, *t* test) for both media, despite the nitrate concentration being reduced to 45% in enBBM_opt_. This indicates the nitrate-specific biomass yield as being a function of the initial nitrate availability, a phenomenon that has recently been recognized and discussed for a fairly comparable *Chlorella* process [27]. Together with an increase in the neutral lipid content from 10.55 ± 0.35% (w w^−1^) to 23.9 ± 1.2% (w w^−1^), this translated into a 2.3-fold increase of volumetric productivity up to 169 ± 7 mg L^−1^ d^−1^.

**Fig. 6:**
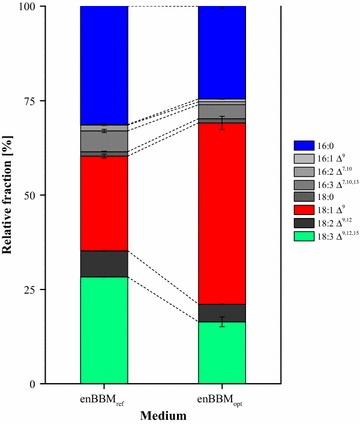
Relative composition of the fatty acids from the neutral lipid product fractions for enBBM_ref_ and enBBM_opt_. Error bars represent min/max from biological replicates (*n* = 2)

Besides the evaluation of productivity-related issues, the relative composition of the fatty acids from the neutral lipid product fraction was compared by gas chromatography time-of-flight mass spectrometry (see Fig. 6).

The obtained fingerprints were in clear agreement with the previous literature reports [69] as palmitic, oleic, linoleic, and α-linolenic acids made up the major product fractions of 85% (enBBM_ref_) and 89% (enBBM_opt_). There are indications that the lipid fingerprint largely depends on cultivation conditions such as temperature [70], illumination [71], etc. However, our results demonstrate that changes in the medium composition can also lead to differences in the fatty acid fingerprint. The fractions of palmitoleic (16:1 Δ^9^), hexadecadienoic (16:2 Δ^7,10^), hexadecatrienoic (16:3 Δ^7,10,13^), stearic (18:0), and linoleic (18:2 Δ^9,12^) remained nearly unchanged. On the contrary, the proportions of palmitic (16:0) and α-linolenic (18:3 Δ^9,12,15^) acids shrank by 22 and 42%, respectively, while linoleic (18:1 Δ^9^) acid increased by 92% to a total share of 48 ± 1.8% using enBBM_opt_. With respect to biodiesel synthesis, this reduction in the polyunsaturated fatty acids’ fraction is clearly advantageous, increasing the fuel’s oxidative stability [72].

### Final medium simplification

In “Kriging-assisted optimization” section, several input variables were identified to be ‘non-relevant’ and thus kept at the respective reference values throughout the whole study. However, for MES and especially for EDTA, a negative, but still non-significant (*p* < 0.05, *t* test) trend was observed. Besides economic aspects, culture media should only contain the necessary ingredients in appropriate concentrations to ensure high nutrient usage efficiency. Thus, an additional variant, in the following denoted as enBBM_opt,min_, was investigated. Here, the concentrations of all ‘non-relevant’ components were set to the respective minimum values during the screening analysis. In particular, this included the complete omissions of MES buffer and the chelator EDTA, as well as NaCl (see Additional file 2 for medium composition), while phosphate availability was reduced to 0.125 × Ref.

In comparison with the results using enBBM_ref_, as well as enBBM_opt_, these adaptations did not change the overall obtained cell dry weight significantly (*p* < 0.05, *t* test) but led to an increase of the neutral lipid to 30.1 ± 1.6% (w w^−1^), while the respective lipid fingerprint remained unchanged in comparison with enBBM_opt_ (see Additional file 11). The resulting volumetric productivity of 212 ± 11 mg L^−1^ d^−1^ represents a total 2.9-fold improvement compared with the reference. Leaving out EDTA and especially the MES buffer drastically reduces the medium costs, so that the price per liter is lowered by 96%. Most probably, MES is not required as the phosphate salts offer sufficient pH stabilization capacity. Although EDTA is commonly used as a metal chelator to improve long-term stability of algae cultivation media, the results clearly indicate that its usage is not beneficial for this specific application. Moreover, the reduction of phosphate concentration to 12.5% is advantageous for large-scale application where the recovery of excess nutrients to prevent overfertilization by wastewater is an important economic aspect. Yet, these results clearly confirm the validity of the initial screening analysis.

### Assessment of achieved volumetric productivity

In the last decade, numerous studies addressed the lipid production of diverse *C. vulgaris* strains in different laboratory-scale batch processes [6, 17, 58, 70, 71, 73–81]. Among these, the average volumetric productivity was approximately 51 ± 36 mg L^−1^ d^−1^ and thus was comparable to the achieved value of 74 ± 1 mg L^−1^ d^−1^ using the enBBM reference medium. However, the reported values exhibit a wide spread, and it has to be assumed that these differences do not only originate from the different strains used, but from process conditions and reactor design as well. Some studies report productivities in the range of 130 mg L^−1^ d^−1^ when cultivating *C.* *vulgaris* in laboratory-scale batch processes with optimized nitrogen availability [17, 80]. Unfortunately, it is not generally clarified if productivities refer to the neutral lipid or the total lipid content. In this study, the volumetric productivity of neutral lipids of up to 212 ± 11 mg L^−1^ d^−1^ clearly exceeds previous reports and thereby underlines the importance of medium optimization not only for nitrate as commonly done, but especially for the concentrations of further salts and trace ions.

## Conclusions

In this study, a blueprint strategy for the accelerated development of phototrophic bioprocesses is presented. This strategy is very efficient in terms of time and material, by incorporating state-of-the-art phototrophic cultivation and analytics with higher throughput that is closely linked to sophisticated experimental design strategies.

Taking neutral lipid production by the unicellular microalga *C. vulgaris* as a model process, the cultivation medium was optimized toward volumetric productivity. Fractional and full factorial designs in combination with Kriging-based approaches for data analysis, visualization, and experimental design allowed for an efficient and effective optimization in terms of time and cost. The optimized process has an approximately threefold increased lipid productivity of 212 ± 11 mg L^−1^ d^−1^, which was achieved with only four experimental rounds with one microtiter plate each.

Besides the commonly addressed concentration of the nitrogen source (here nitrate), especially magnesium, calcium, and various trace elements were shown to be of crucial importance. Analysis tools furthermore revealed multi-parameter interactions that could have been overlooked otherwise. Over and above this, the concentration of non-relevant medium components was successfully minimized, contributing to reducing medium cost. Taking all the above results together, a smart combination of microscale phototrophic cultivation with sophisticated design of experiments led to a tremendous improvement of neutral lipid production with *C. vulgaris*, at the same time reducing cost for media components by 96%, while all other process performance indicators were kept constant.

## Additional files

**Additional file 1.** Fractional factorial design for the initial screening analysis and corresponding measurement data of the individual cultivations.

**Additional file 2.** Medium composition of enBBM_ref_, enBBM_opt_ and enBBM_opt,min_.

**Additional file 3.** Estimated effect of two factor interactions with MgSO_4_. Estimations are based on the experiments in section “Fractional factorial” using the fractional factorial design and the corresponding measurement data given in Additional material 1.

**Additional file 4.** Experimental design and corresponding measurement data evaluated in section “Full factorial”.

**Additional file 5.** Screening plot around reference point (enBBM_ref_ medium). Estimation of the functional relationship between media components and lipid productivity was done by Kriging. The Kriging model is based on the experiments in section “Kriging-assisted optimization” using the open source software KriKit.

**Additional file 6.** Experimental design and corresponding measurement data evaluated in section “Locating optimal medium composition”.

**Additional file 7.** Analysis of calcium precipitation by means of optical density measurements. Shaded area represents the parameter space covered during cultivation experiments. Error bars deviated from technical replicates (n = 3).

**Additional file 8.** Measured data and Kriging interpolation after the third round of experiments in three-dimensional representation. The non-varied components were fixed at MgSO_4_=1.7xRef, CaCl_2_=0.82xRef, Trace=1xRef, or NaNO_3_=0.37xRef. Red dots indicate measurement data.

**Additional file 9.** Experimental design and corresponding measurement data evaluated in section “Refining the optimum”.

**Additional file 10.** Measurement data and updated Kriging interpolation after the fourth round of experiments in three-dimensional representation. The non-varied components were fixed at MgSO_4_=1.7xRef, CaCl_2_=0.82xRef, Trace=1xRef, or NaNO_3_=0.37xRef. Red dots indicate measurement data.

**Additional file 11.** Relative composition of the fatty acids from the neutral lipid product fraction for enBBM_opt_ and enBBM_opt,min_. Error bars represent min/max from biological replicates (n = 2).

## Abbreviations

DoE: Design of Experiments
EI: Expected Improvement
enBBM: enriched Bold’s Basal Medium
KriKit: Kriging toolKit
MCMC: Markov Chain Monte Carlo
nLC [% (w w^−1^)]: neutral lipid content
OD_750_ [−]: optical density at 750 nm
P_vol_ [mg L^−1^ d^−1^]: volumetric productivity
Ref: reference value
µ [d^−1^]: exponential growth rate
