# Supplementary figures and images for: A framework for accelerated phototrophic bioprocess development: integration of parallelized microscale cultivation, laboratory automation and Kriging-assisted experimental design
Source: Biotechnol Biofuels. 2017 Jan 31;10:26. doi: 10.1186/s13068-017-0711-6 (PMC5282810; doi:10.1186/s13068-017-0711-6)

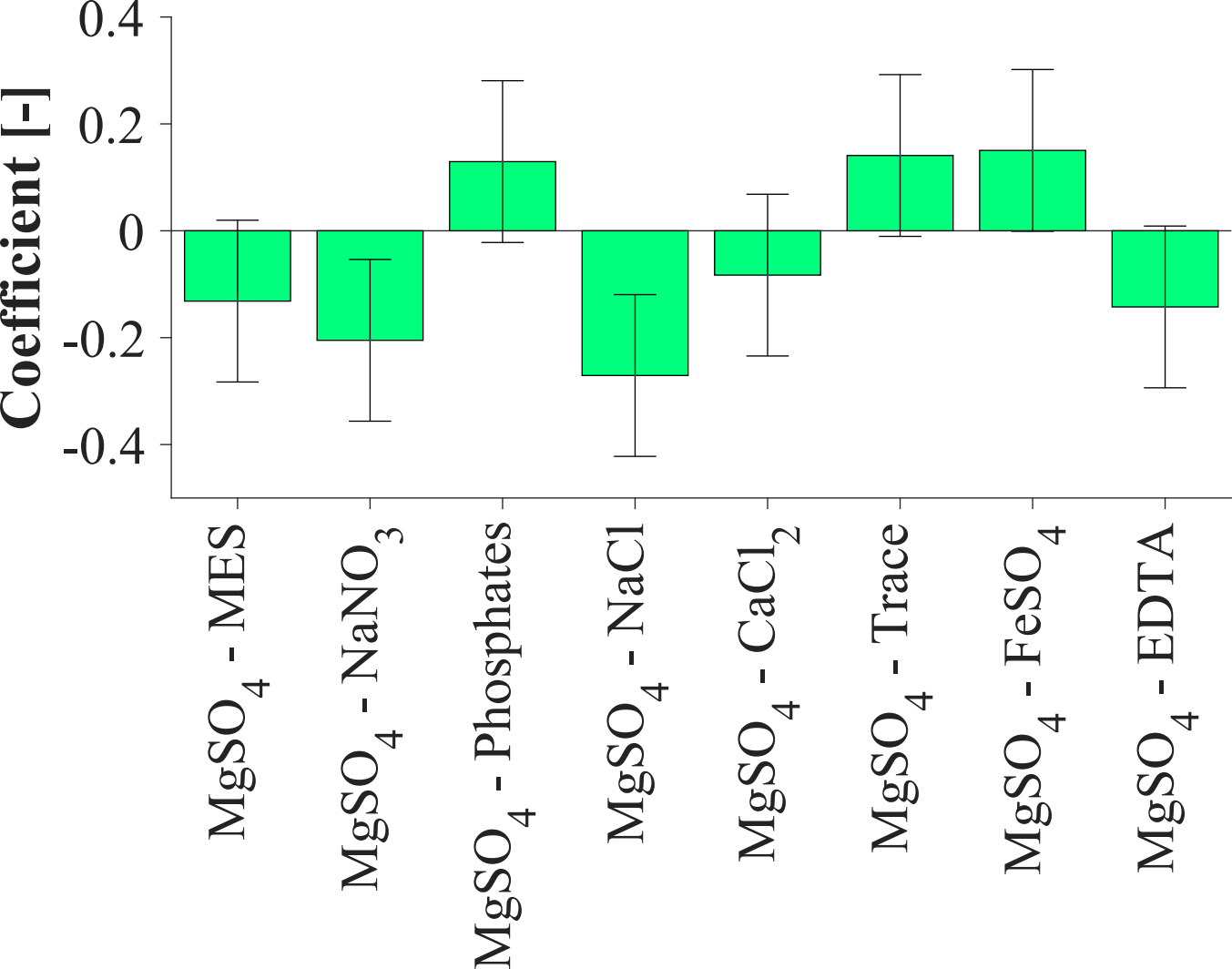

Supplement: Supplementary file 3 — Additional file 3. Estimated effect of two factor interactions with MgSO4. Estimations are based on the experiments in section “Fractional factorial” using the fractional factorial design and the corresponding measurement data given in Additional material 1. [file 13068_2017_711_MOESM3_ESM.pdf]

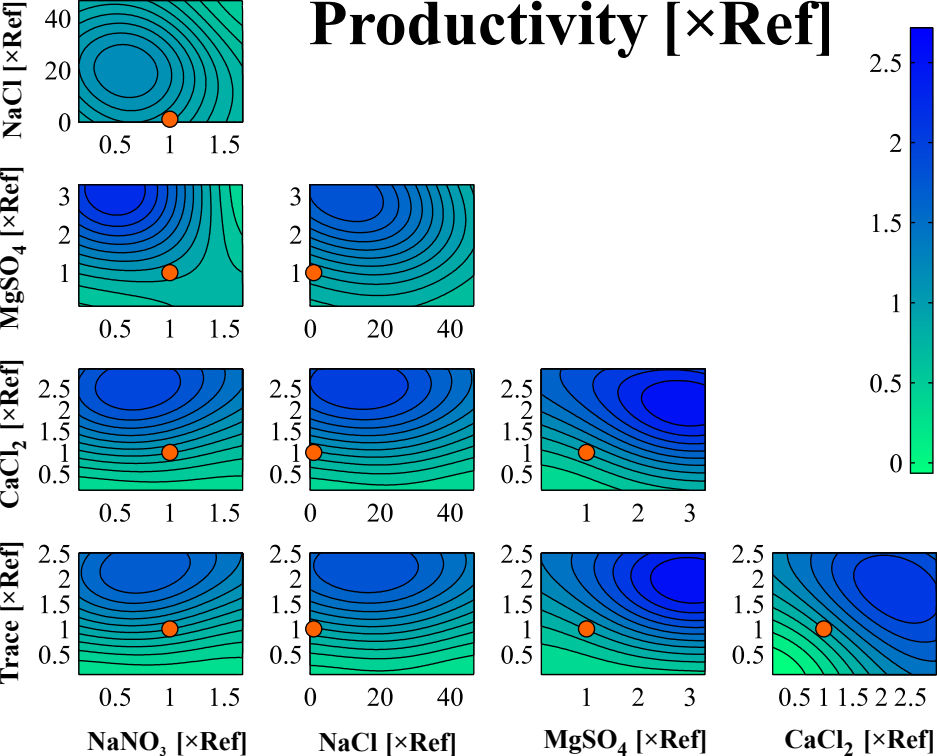

Supplement: Supplementary file 5 — Additional file 5. Screening plot around reference point (enBBMref medium). Estimation of the functional relationship between media components and lipid productivity was done by Kriging. The Kriging model is based on the experiments in section “Kriging-assisted optimization” using the open source software KriKit. [file 13068_2017_711_MOESM5_ESM.pdf]

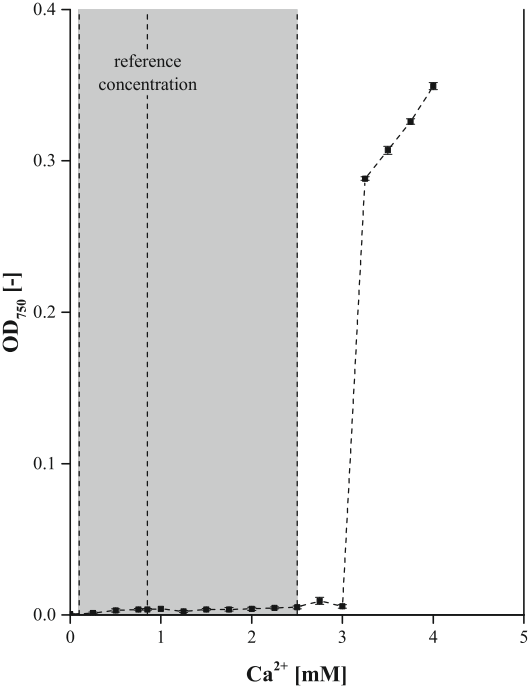

Supplement: Supplementary file 7 — Additional file 7. Analysis of calcium precipitation by means of optical density measurements. Shaded area represents the parameter space covered during cultivation experiments. Error bars deviated from technical replicates (n = 3). [file 13068_2017_711_MOESM7_ESM.pdf]

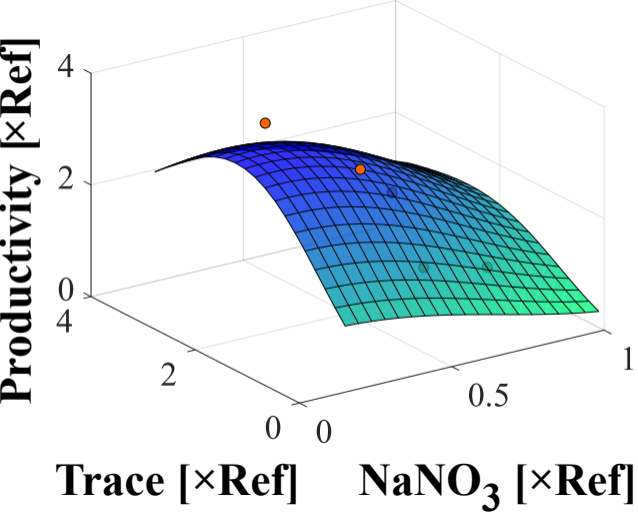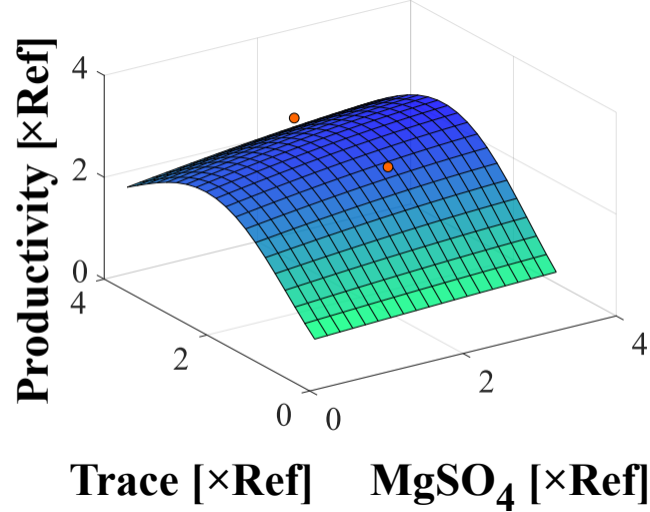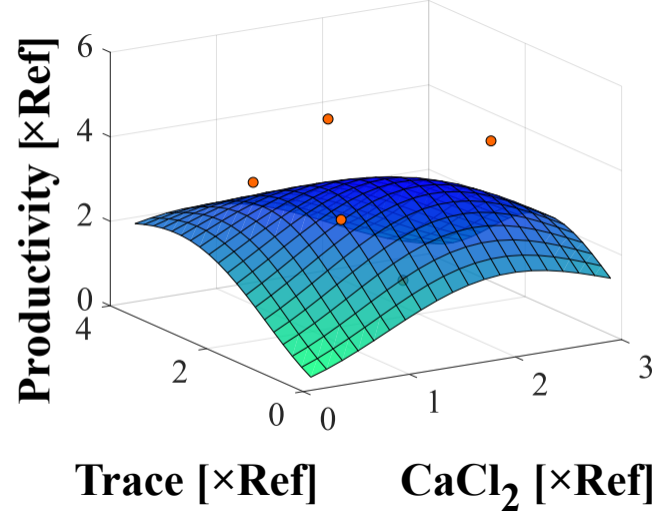

Supplement: Supplementary file 8 — Additional file 8. Measured data and Kriging interpolation after the third round of experiments in three-dimensional representation. The non-varied components were fixed at MgSO4=1.7xRef, CaCl2=0.82xRef, Trace=1xRef, or NaNO3=0.37xRef. Red dots indicate measurement data. [file 13068_2017_711_MOESM8_ESM.pdf]

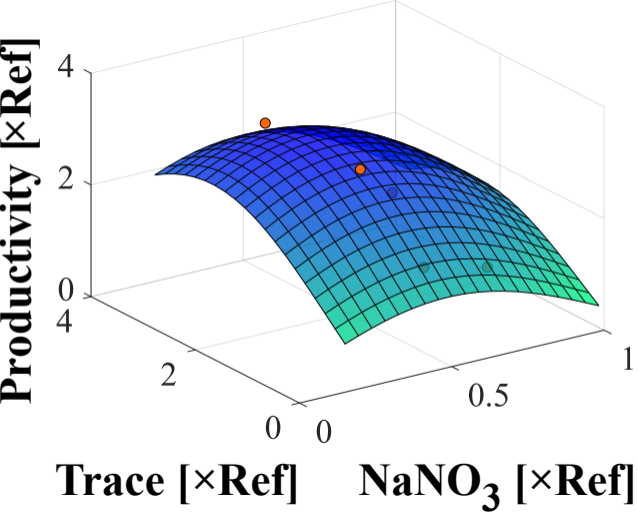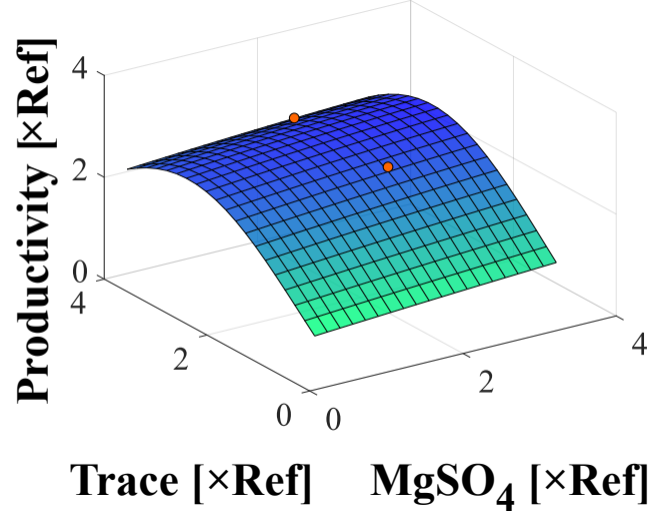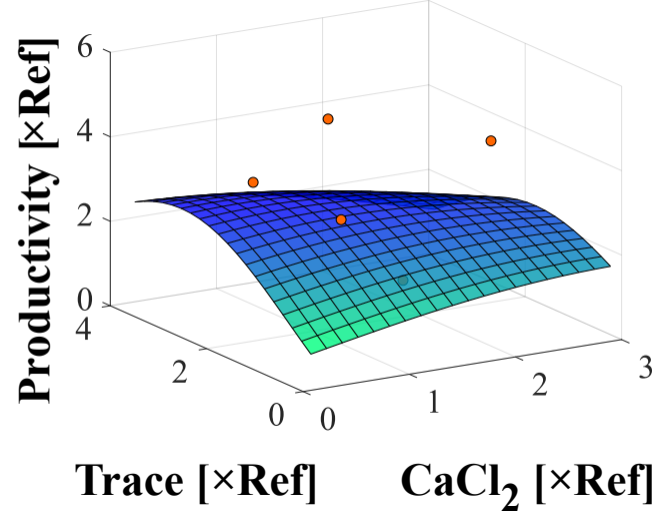

Supplement: Supplementary file 10 — Additional file 10. Measurement data and updated Kriging interpolation after the fourth round of experiments in three-dimensional representation. The non-varied components were fixed at MgSO4=1.7xRef, CaCl2=0.82xRef, Trace=1xRef, or NaNO3=0.37xRef. Red dots indicate measurement data. [file 13068_2017_711_MOESM10_ESM.pdf]

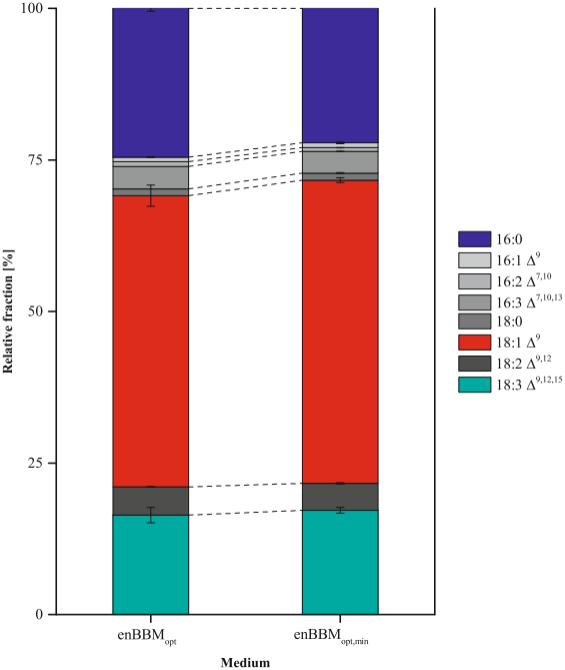

Supplement: Supplementary file 11 — Additional file 11. Relative composition of the fatty acids from the neutral lipid product fraction for enBBMopt and enBBMopt,min. Error bars represent min/max from biological replicates (n = 2). [file 13068_2017_711_MOESM11_ESM.pdf]
